# Myocyte enhancer factor 2A delays vascular endothelial cell senescence by activating the PI3K/p-Akt/SIRT1 pathway

**DOI:** 10.18632/aging.102015

**Published:** 2019-06-10

**Authors:** Benrong Liu, Lin Wang, Wenyi Jiang, Yujuan Xiong, Lihua Pang, Yun Zhong, Chongyu Zhang, Wenchao Ou, Chaowei Tian, Xiaohui Chen, Shi-Ming Liu

**Affiliations:** 1 Guangzhou Institute of Cardiovascular Disease, Guangdong Key Laboratory of Vascular Diseases, State Key Laboratory of Respiratory Disease, The Second Affiliated Hospital, Guangzhou Medical University, Guangzhou 510260, P. R. China; 2 Department of Laboratory Medicine, The Second Affiliated Hospital of Guangzhou University of Chinese Medicine, Guangzhou 510220, P. R. China; 3 Department of Emergency, The Second Affiliated Hospital, Guangzhou Medical University, Guangzhou 510260, P. R. China

**Keywords:** myocyte enhancer factor 2A, Phosphatidylinositol-4,5-bisphosphate 3-kinase catalytic subunit alpha (PIK3CA), Phosphatidylinositol-4,5-bisphosphate 3-kinase catalytic subunit gamma (PIK3CG), Sirtuin 1 (SIRT1), senescence

## Abstract

Myocyte enhancer factor 2A (MEF2A) dysfunction is closely related to the occurrence of senile diseases such as cardiocerebrovascular diseases, but the underlying molecular mechanism is unclear. Here, we studied the effects of MEF2A on the senescent phenotype of vascular endothelial cells (VEC) and downstream signaling pathway, and the association between plasma MEF2A levels and coronary artery disease (CAD). Results showed that MEF2A silencing promoted cell senescence and down-regulated PI3K/p-AKT/Sirtuin 1 (SIRT1) expression. MEF2A overexpression delayed cell senescence and up-regulated PI3K/p-AKT/SIRT1. Hydrogen peroxide (H_2_O_2_) treatment induced cellular senescence and down-regulated the expression of MEF2A and PI3K/p-AKT/SIRT1. MEF2A overexpression inhibited cellular senescence and the down-regulation of PI3K/p-AKT/SIRT1 induced by H_2_O_2_. Further study revealed that MEF2A directly up-regulated the expression of PIK3CA and PIK3CG through MEF2 binding sites in the promoter region. Pearson correlation and logistic regression analysis showed that the plasma level of MEF2A was negatively correlated with CAD, and with age in the controls. These results suggested that MEF2A can directly up-regulate PI3K gene expression, and one of the molecular mechanisms of delaying effect of MEF2A on VEC cell senescence was SIRT1-expression activation through the PI3K/p-Akt pathway. Moreover, the plasma MEF2A levels may be a potential biomarker for CAD risk prediction.

## INTRODUCTION

Myocyte enhancer factor 2 (MEF2) family shares a 58-amino acid homology domain that benefits DNA binding and dimerization. MEF2 belongs to the MADS-box super family and is a class of key transcriptional regulators that positively regulate cell differentiation, cell proliferation, morphogenesis, cell survival, and apoptosis [1, 2]. MEF2A is one of the members of the MEF2 family and participates in various cellular processes, including muscle development, neuronal differentiation, cell-growth control, and apoptosis, in the form of homodimers or heterodimers [3]. As a very important transcription factor, MEF2A has an essential DNA-binding site in the MyoDa gene-control region and can activate many muscle-specific, growth factor-induced, and stress-induced genes [4, 5]. The key roles of MEF2A in skeletal- and cardiac-muscle development have been demonstrated in several studies. Naya et al. [6] found that MEF2A knockout mice die suddenly in the first week after birth and exhibit pronounced right ventricle dilation, myofibrillar fragmentation, mitochondrial disorganization, and activation of fetal cardiac gene program. Chen et al. [7] reported that MEF2A is involved in the process of AKT2-mediated cardiomyocyte development. A comprehensive analysis of target genes for MEF2 in cardiac and skeletal muscles has revealed that MEF2A is involved in the regulation of growth and proliferation gene networks in addition to regulating gene networks involved in muscle development [8].

Wang et al. [9] first revealed in 2003 that the 21-bp deletion mutation in exon 11 of MEF2A gene is co-segregated with premature coronary artery disease (p-CAD) in a family lineage. Since then, many researchers have extensively studied the correlation between MEF2A genetic variation and coronary artery disease (CAD). However, the results were disappointing as no genetic variation tightly associated with CAD has been found in the MEF2A gene region [10–16]. Recently, a novel 6-bp deletion in the 11^th^ exon of MEF2A has been found to be closely related to early-onset CAD in a Chinese family [17]. However, whether the novel 6-bp deletion is also a rare mutation in the population, similar to the 21-bp deletion found previously, is unknown. Thus, this finding has little value in predicting CAD risk and early diagnosis. Kim et al. [18] demonstrated that the activities of MEF2A and MEF2C in pulmonary artery endothelial cells from patients with pulmonary hypertension (PAH) is significantly impaired, and that the recovery of MEF2A and MEF2C can rescue the cellular phenotype from PAH. In apoE-deficient mice, MEF2A inhibition accelerates atherosclerosis [19]. These findings suggest that decreased expression or dysfunction of MEF2A play important roles in the pathogenesis of vascular disease.

The development of vascular disease is closely related to VEC aging, and many genes associated with aging or longevity play important roles in maintaining vascular function [20]. However, the functions of MEF2A in vascular aging and cellular senescence remain to be explored. Some studies have suggested that MEF2A is involved in cellular senescence or vascular aging. For example, Zhao et al. [21] showed that MEF2A can promote H_2_O_2_-induced smooth-muscle-cell senescence. The roles of MEF2 in promoting the proliferation of endothelial cells and endothelial progenitor cells, as well as in maintaining the normal function of endothelial cells, have also been elucidated [22, 23]. MEF2A can also reportedly prevent apoptosis or aging by regulating cellular respiratory chain-related molecules participating in cellular energy metabolism [24]. However, few studies provide direct evidence of the association of MEF2A expression changes or dysfunction with cellular senescence.

Our early studies have shown that the inhibition of MEF2A expression in human coronary endothelial cells accelerates cellular senescence and down-regulates PIK3CG gene expression [25]. Accordingly, we became curious about possible MEF2A involvement in vascular endothelial cell (VEC) aging by regulating the PI3K pathway, and whether the plasma levels of MEF2A can indicate risk of cardiovascular diseases such as CAD. In the present study, we performed a series of experiments in human umbilical-vein endothelial cells (HUVECs) to elucidate the regulatory mechanism of MEF2A on PI3K and the roles and molecular mechanism of MEF2A in cell senescence. Results indicated that MEF2A delayed cell senescence by positively regulating the PI3K/p-Akt/Sirtuin 1 (SIRT1) signaling pathway, inhibited oxidative stress-induced cellular senescence, and directly positively regulated PI3K gene expression. The decreased plasma level of MEF2A was also significantly associated with CAD risk.

## RESULTS

### Changes in MEF2A expression affected the senescent phenotypes of HUVECs

**Figure 1:**
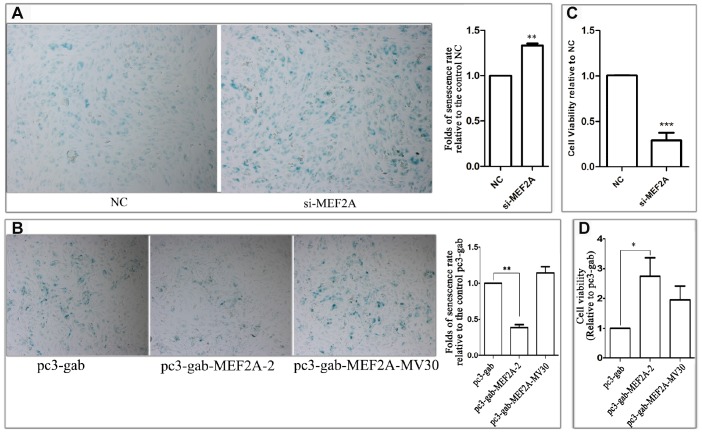
**The effect of changes in the expression of MEF2A on the HUVEC phenotype.** (**A**) The images show the SA-β-galactosidase staining positive cells (blue) in NC and MEF2A-specific siRNA (si-MEF2A) group, and the histogram shows the fold changes of the senescence rate relative to NC group. (**B**) The images show the SA-β-galactosidase staining positive cells in control plasmid (pc3-gab), wild type MEF2A overexpression plasmid (pc3-gab-MEF2A-2) and frameshift mutant MEF2A plasmid (pc3-gab-MEF2A-MV30) group, and the histogram shows the fold changes of the senescence rate relative to pc3-gab group. (**C**) Fold changes of cell viability relative to NC group. (**D**) Fold changes of cell viability relative to pc3-gab group. The number of total cells and SA-β-gal staining positive cells were counted in 20-30 images photographed from different fields of view in the duplicate wells in each experiment. The senescence rate was calculated by dividing the number of SA-β-gal staining positive cells by the number of the total cells. The histograms show the mean ± SEM of more than three independent experiments. *, *P* < 0.05; **, *P* < 0.01; ***, *P* < 0.001.

To observe the effect of MEF2A expression on endothelial-cell senescence phenotype, MEF2A-specific siRNA and MEF2A overexpression plasmid were transfected into HUVECs, respectively. About 72-96 h after transfection, SA-β-gal staining was performed and the staining-positive cells were counted. Results showed that the percentage of SA-β-gal staining-positive cells in the siRNA interference group was significantly higher than that in the negative control group (Figure 1A), whereas the percentage of senescent phenotype cells in MEF2A overexpression group was significantly lower than that in the empty plasmid group and the MEF2A mutant group (pc-gab-MEF2A-MV30) (Figure 1B), pc-gab-MEF2A-MV30 is a non-functional control vector encoding a MEF2A transcript with a frameshift mutation. The inhibition of MEF2A expression in HUVECs significantly reduced cell viability (Figure 1C), whereas MEF2A overexpression in HUVECs significantly increased cell viability (Figure 1D). These results indicated that the inhibition of MEF2A expression reduced cell viability and enhanced the cellular aging phenotype of HUVECs, whereas MEF2A overexpression increased cell viability and reduced cellular-senescence phenotype.

### MEF2A up-regulated the PI3K/p-Akt/SIRT1 signaling pathway

**Figure 2:**
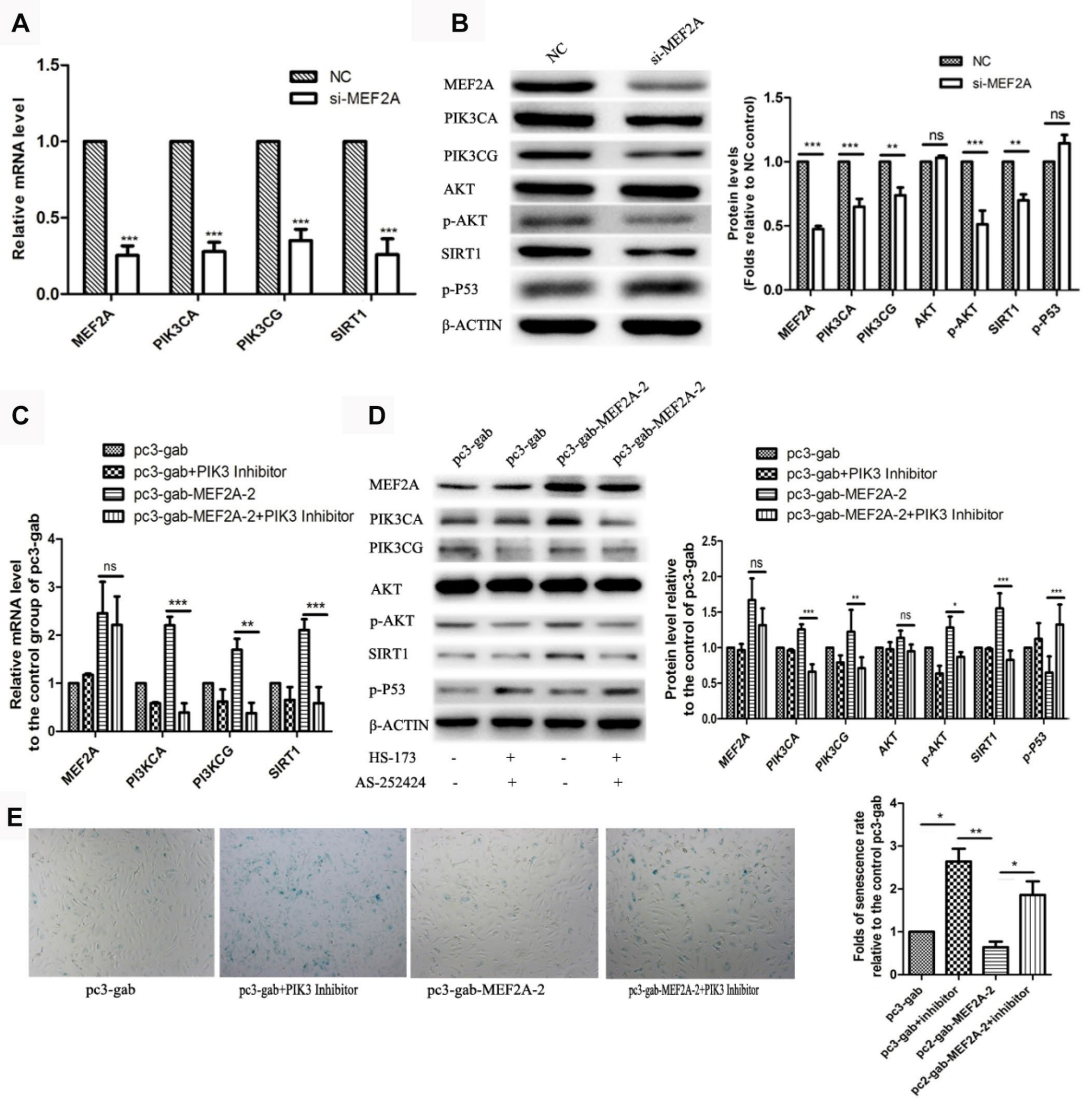
**The influence of changes in expression of MEF2A on downstream gene expression.** (**A**) Changes in downstream gene mRNA levels after transfection of si-MEF2A in HUVEC; (**B**) Changes in downstream gene protein levels after transfection of si-MEF2A in HUVEC; (**C**) Effect of transfection of MEF2A overexpression plasmid or transfection of MEF2A overexpression plasmid plus PI3K inhibitor on the mRNA level of downstream gene in HUVEC; (**D**) Influence of transfection of MEF2A overexpression plasmid or transfection of MEF2A overexpression plasmid plus PI3K inhibitors on downstream gene protein levels in HUVEC. (**E**) Impact of transfection of MEF2A overexpression plasmid or transfection of MEF2A overexpression plasmid plus PI3K inhibitor on HUVEC phenotype. The mRNA level, protein level and senescence rate were expressed as the mean fold changes relative to the control group, and the error bars represent the standard error of the fold changes in 3 independent experiments. *, *P* < 0.05; **, *P* < 0.01; ***, *P* < 0.001; ns, no significance.

The PI3K/p-Akt/SIRT1 signaling pathway plays an important role in regulating cell proliferation, cell survival, development, cell senescence, and other biological processes. To understand the molecular mechanism of the effect of MEF2A on cellular senescence, real-time fluorescent quantitative PCR and immune-blotting were used to detect the effect of inhibition or MEF2A overexpression on the expression of several key genes in the PI3K/p-Akt/SIRT1 signaling pathway. When MEF2A was knocked down in HUVECs, the mRNA levels of PIK3CA, PIK3CG, and SIRT1 decreased significantly compared with the negative control group (Figure 2A). Western blotting showed that PIK3CA, PIK3CG, SIRT1, and p-AKT levels were down-regulated with the inhibition of MEF2A, and the P53 level was up-regulated (Figure 2B). Conversely, PIK3CA, PIK3CG, and SIRT1 mRNA levels were significantly up-regulated in the MEF2A-overexpression HUVECs (Figure 2C), and the protein levels of PIK3CA, PIK3CG, SIRT1, and p-Akt notably increased but P53 decreased (Figure 2D). When the specific inhibitors of PIK3CA and PIK3CG were added while overexpressing MEF2A, the activities of p-Akt and P53 did not change with increased MEF2A, and SIRT1 expression did not increase with increased MEF2A (Figure 2C and 2D). This result suggested that MEF2A up-regulated the expression of the downstream gene SIRT1 by positively regulating the expression of PIK3CA and PIK3CG genes. Compared with the empty vector group, the transfection of MEF2A expression plasmid decreased the proportion of the SA-β-gal staining-positive cells, but when PI3K inhibitor was added, it significantly enhanced SA-β-gal staining and eliminated the effect of MEF2A overexpression on HUVECs (Figure 2E). This result implicated that MEF2A delayed HUVEC aging by activating PI3K/p-Akt/SIRT1 pathway.

### Down-regulation of MEF2A may be one of the mechanisms underlying oxidative-stress-induced senescence in HUVECs

**Figure 3:**
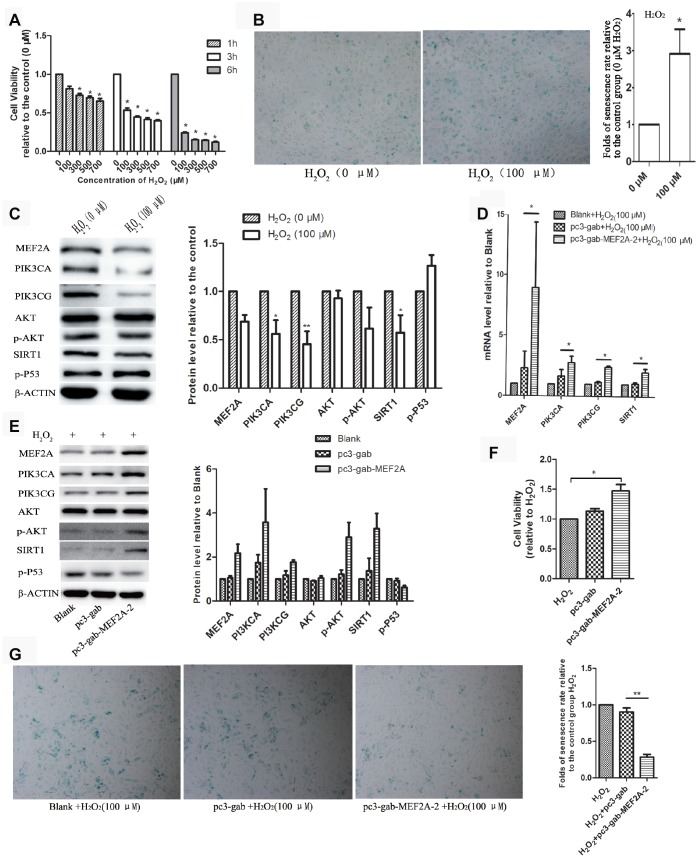
**The role of MEF2A in hydrogen peroxide-induced cell senescence.** (**A**) Effects of different concentrations of H_2_O_2_ on the viability of HUVEC cells at different time. (**B**) Impact of treatment with 100 μM H_2_O_2_ on the senescent phenotype of HUVEC for 1 hour. (**C**) Influence of treatment with 100 μM H_2_O_2_ for 1 hour on gene protein levels in HUVEC. (**D**) Changes in mRNA levels of genes of interest in each group. (**E**) Changes in protein levels of genes of interest in each group. (**F**) Changes in cell viability of each group. (**G**) Changes in the cellular senescence phenotype of each group. The cell viability, senescence rate, protein level and mRNA level were expressed as the mean fold changes relative to the control group, and the error bars represent the standard error of the fold changes in 3 independent experiments. Blank: HUVEC transfected without plasmids; pc3-gab: HUVEC transfected with empty vector (pc3-gab); pc3-gab-MEF2A-2: HUVEC transfected with MEF2A overexpression plasmid. *, *P* < 0.05; **, *P* < 0.01; ***, *P* < 0.001.

Oxidative stress is one of the main factors inducing cell senescence. Cellular senescence is accelerated when cells are exposed to oxidative stress factors such as hydrogen peroxide in vitro. In this study, we observed that the treatment of HUVECs with H_2_O_2_ showed a decrease in cell viability in concentration- and time-dependent manners (Figure 3A). In the subsequent experiments, we treated the cells with 100 μM H_2_O_2_ for 1 h. Results showed that the treatment of HUVECs with 100 μM H_2_O_2_ for 1 h significantly accelerated cell senescence, decreased cell viability (Figure 3B), significantly down-regulated the expression of MEF2A and the downstream genes: PI3K, p-AKT and SIRT1, and increased the expression of P53 (Figure 3C). When MEF2A was overexpressed in HUVECs and then treated with 100 μM H_2_O_2_, compared with the control groups, MEF2A overexpression inhibited the H_2_O_2_-induced changes in the PI3K/p-AKT/SIRT1 and P53 levels (Figure. 3D and 3E), rescued H_2_O_2_-induced cellular senescence, and increased cell viability (Figure 3F and 3G). Thus, the down-regulation of MEF2A expression may be one of the mechanisms by which H_2_O_2_ induced senescence in HUVECs.

### MEF2A directly regulated the expression of PIK3CA and PIK3CG genes

**Figure 4:**
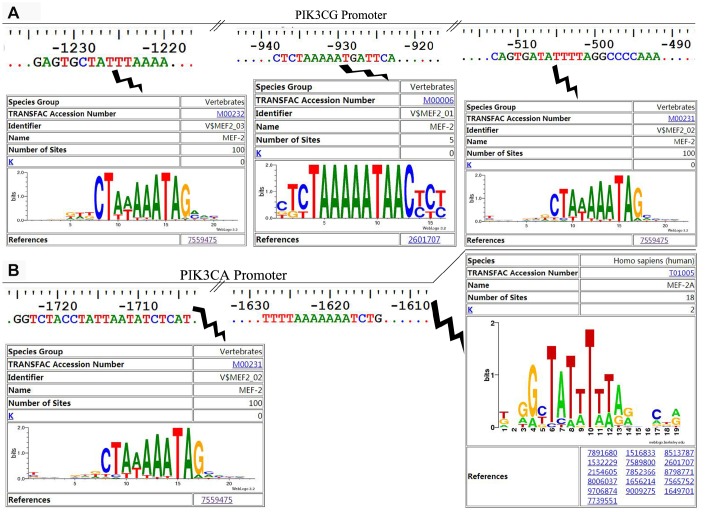
**The potential MEF2 binding sites in the proximal promoter region of PIK3CA and PIK3CG predicted by LASAGNA-Search 2.0.** (**A**) Sequence characteristics and the location of the potential binding sites of MEF2 in the proximal promoter region of PIK3CG; (**B**) Sequence characteristics and the location of the potential binding sites of MEF2 in the proximal promoter region of PIK3CA.

**Figure 5:**
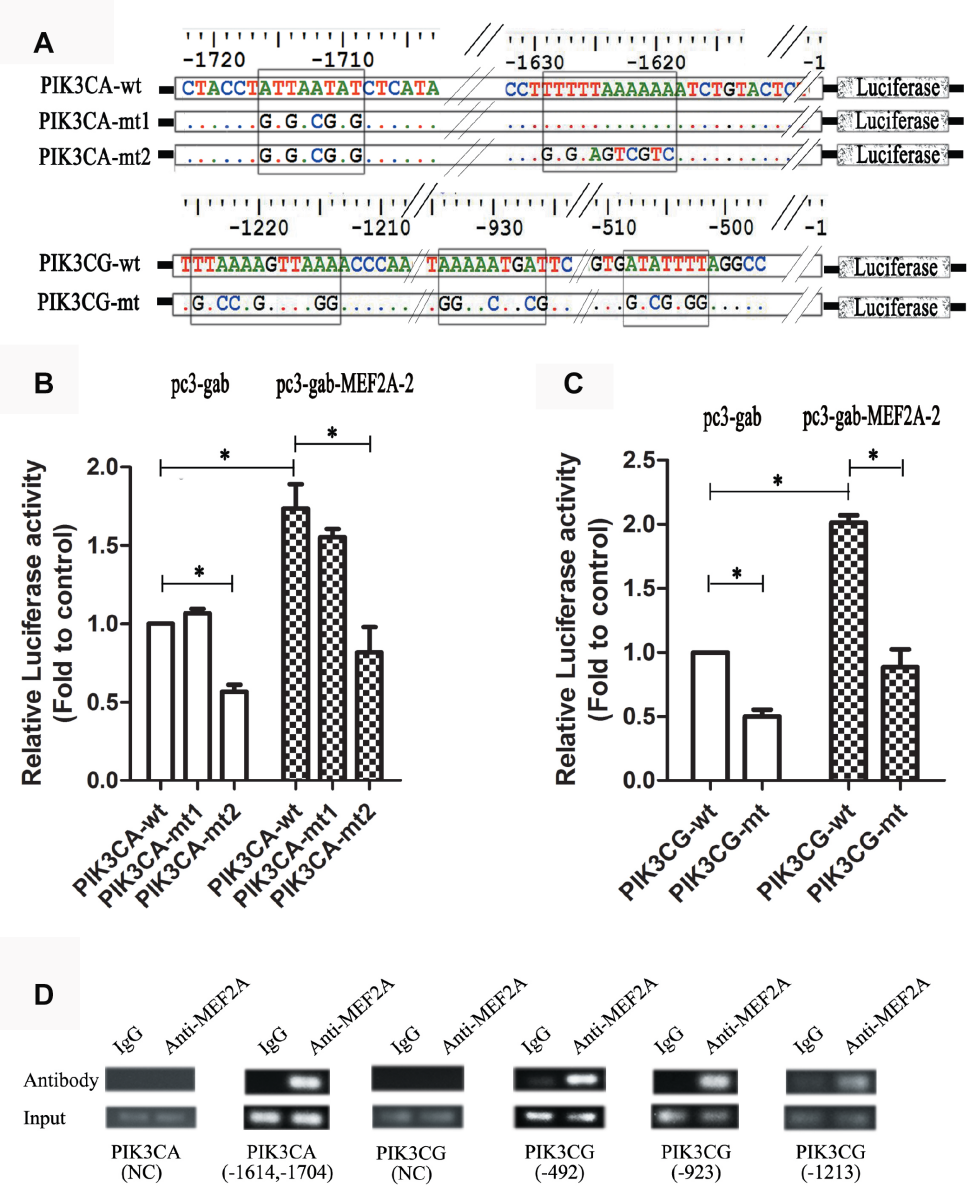
**Dual luciferase reporter gene assay and ChIP assay.** (**A**) Strategies for the construction of PIK3CA and PIK3CG promoter vectors including wild type and mutant type (the predicted MEF2 binding sites were mutated). (**B** and **C**) PIK3CA and PIK3CG promoter vectors (wild and mutant) were cotransfected into 293T cells with the empty expression vector (pc3-gab) or the overexpression vector of MEF2A (pc3-gab-MEF2A-2), respectively. (**D**) Chromosome immunoprecipitation assay. NC: Negative control, the PCR primers were designed to be located away from the MEF2 binding site. The numbers indicated the position of the predicted MEF2 binding sites in the promoter region. *, *P* < 0.05, indicates a statistically significant difference.

The online software LASAGNA-search 2.0 (http://biogrid-lasagna.engr.uconn.edu/lasagna_search/) was used to predict the potential MEF2 binding sites in the promoter region of PIK3CG and PIK3CA from 2000 bp upstream to 200 bp downstream of the transcription start site. Results showed potential MEF2 binding sites at -1628-(-1614) bp and -1725-(-1704) bp in the promoter region of PIK3CA (Figure 4A), and at -513-(-492) bp, -938-(-923) bp and -1234-(-1220) bp in the promoter region of PIK3CG (Figure 4B). The wild-type promoter vectors containing the above potential MEF2 binding sites and the mutant promoter vectors mutated with all the above-mentioned potential MEF2 binding sites were constructed for PIK3CA and PIK3CG (Figure 5A). PIK3CA and PIK3CG wild-type promoter vectors and mutant promoter vectors were co-transfected, respectively, into 293T cells with MEF2A overexpression plasmid or the negative control vector pc3-gab, and luciferase activity was analyzed. Results showed that the luciferase activity of the PIK3CA and PIK3CG wild-type promoter vectors was conspicuously higher than that of the PIK3CA and PIK3CG mutant promoter vectors (PIK3CA-mt2 and PIK3CG-mt) whether in the control group (pc-gab) or in the MEF2A overexpression group (pc-gab-MEF2A-2 group) (Figure 5B and 5C). These results suggested that MEF2A may directly regulate the expression of PIK3CG and PIK3CA genes by interacting with the potential MEF2 binding sites in their promoter region. PIK3CA-mt1 mutated the site only at -1725bp-(- 1704) bp, and its promoter activity was similar to that of the wild-type promoter vector (PIK3CA-wt). PIK3CA-mt2 mutated the both sites at -1725bp-(- 1704) bp and -1628-(-1614) bp, and its promoter activity was much lower than that of PIK3CA-mt1 and PIK3CA-wt (Figure 5A and 5B). These results indicated that the site at -1628- (-1614) bp in the PIK3CA promoter region is very important for MEF2A to activate PIK3CA expression.

ChIP assay showed strong binding between MEF2A and the PIK3CA promoter region at -1614 to -1704 bp, as well as the PIK3CG promoter region at -513-(-492) bp, -938-(-923) bp, and -1234-(-1220) bp (Figure 5D). This result indicated that MEF2A directly regulated the transcription of PIK3CA and PIK3CG.

### Decrease in plasma MEF2A level increased the risk of CAD

**Figure 6:**
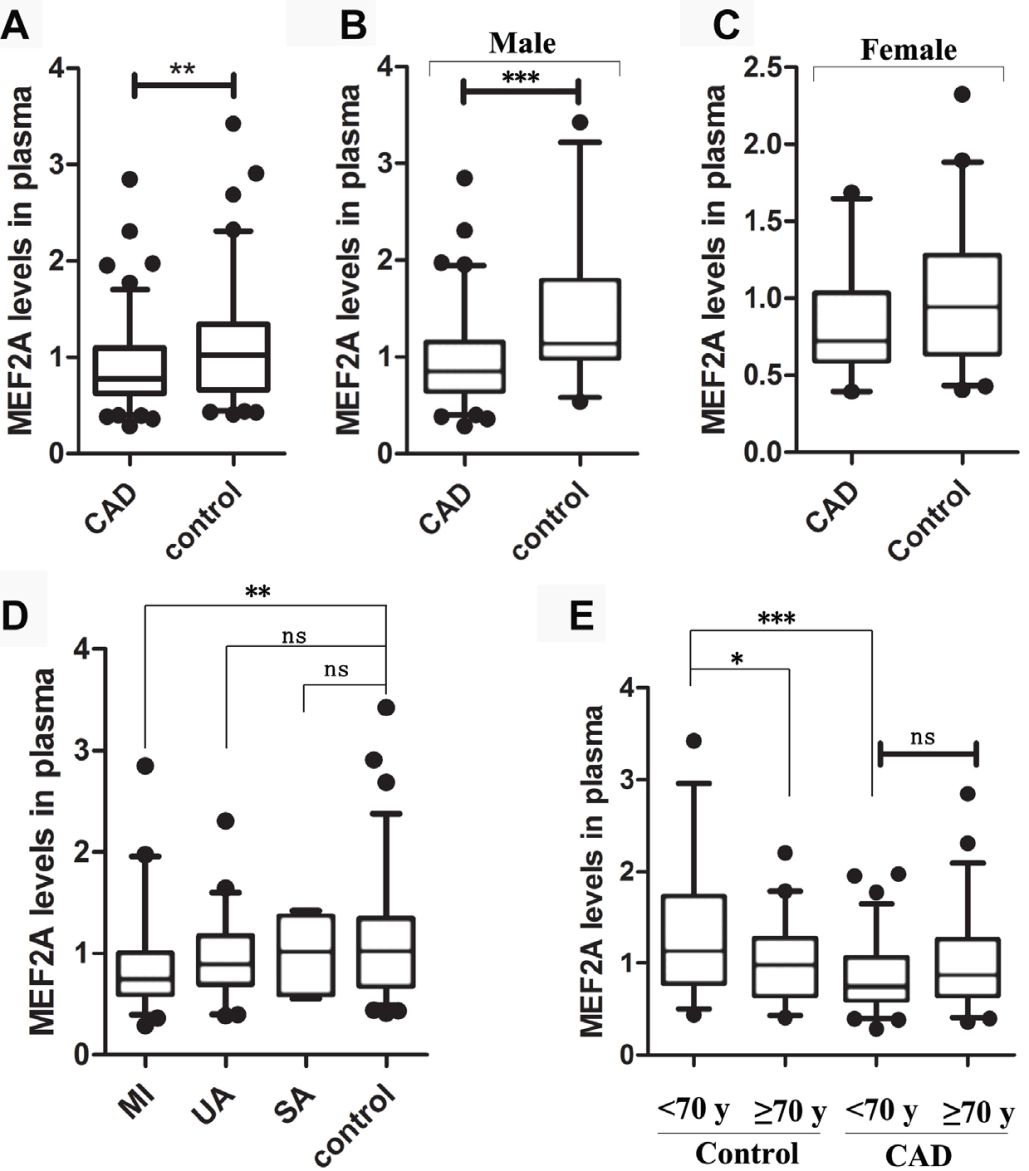
**The statistical distribution of the plasma level of MEF2A in different groups or sub-groups.** (**A**) Plasma MEF2A levels in CAD group and normal control group. (**B**) Plasma MEF2A levels in male CAD subgroup and male control subgroup. (**C**) Plasma MEF2A levels in female CAD subgroup and female control subgroup. (**D**) Plasma levels of MEF2A in the subgroups: myocardial infarction subgroup (MI), unstable angina pectoris subgroup (UA), stable angina pectoris subgroup (SA), and in the controls. (**E**) Plasma levels of MEF2A in the subgroups categorized by age. The statistical distribution was presented with Box and whisker plot. The horizontal lines within the boxes represent the median value and the vertical lines extending below and above the boxes indicate 5-95% percentile values, respectively. The significant difference between any two groups or subgroups was tested by using Wilcoxon rank sum test. *, *P* < 0.05; **, *P* < 0.01; ***, *P* < 0.001.

To reveal whether plasma MEF2A levels decreased with age or were associated with the risk of cardiovascular disease, we measured plasma MEF2A levels in individuals in the CAD and normal control groups. Baseline information for the two groups is detailed in Table 1, and details of information for grouping and for plasma MEF2A level in each group or subgroup are shown in Table 2. The statistical distribution of the plasma level of MEF2A in different groups/sub-groups was presented with a box and whisker plot (whiskers: 5-95% percentile) (Figure 6). Results showed that the plasma level of MEF2A in the CAD group (0.91 ± 0.42 ng/ml) was significantly lower than that in the control group (1.14 ± 0.58 ng/ml) (*z* = -2.939, *P* = 0.003) (Table 2, Figure 6A). A significant difference existed in the gender ratio between the two groups, so to eliminate the impact of the discrepancy in gender ratio on the analysis results, we further divided the CAD and control groups into subgroups according to gender identity and disease status. Results showed that the plasma level of MEF2A in the male CAD subgroup (0.95 ± 0.45 ng/ml) was significantly lower than that in the male control subgroup (1.42 ± 0.72 ng/ml) (*z*= -3.493, *P* < 0.001) (Table 2, Figure 6B), and the plasma level of MEF2A in the female CAD subgroup (0.83 ± 0.34 ng/ml) was also visibly lower than that in the female control subgroup (0.98 ± 0.42 ng/ml) (*z* = -1.797, *P* = 0.072) (Table 2, Figure 6C). We also divided the CAD samples into acute myocardial infarction subgroup (MI), unstable angina subgroup (UA), and stable angina subgroup (SA) according to the severity of the disease. Results showed that a gradually decreased plasma level of MEF2A was associated with an increased severity of disease (Figure 6D). To observe the association between plasma levels of MEF2A and age, the normal control group and CAD group were divided into <70 year-old subgroup (<70y-Con and <70y-CAD) and ≥70 year-old subgroup (≥70y-Con and ≥70y-CAD), respectively (70 years old was selected as the dividing line of subgroup after considering the close number of samples included in the corresponding subgroups). Results showed that the plasma MEF2A level in the <70y-Con subgroup (1.31 ± 0.69 ng/ml) was significantly higher than that in the ≥70y-Con subgroup (0.98 ± 0.40 ng/ml) (*z* = -2.104, *P* = 0.035) and in the <70y-CAD subgroup (0.85 ± 0.37 ng/ml) (*z* = -3.816, *P* < 0.001), but no significant difference was observed in the plasma MEF2A level between the <70y-CAD and ≥70y-CAD subgroups (Table 2, Figure 6E).

**Table 1:** Baseline characters of samples from CAD group and normal control group.

**Indicator**	**CAD (n=118)**	**Control (n=76)**	***P value***
Male/female	80/38	27/49	*< 0.001^a^*
Smoking/no smoking	56/62	12/54	*< 0.001^a^*
Diabetes/no diabetes	12/106	25/51	*< 0.001^a^*
Age (years, mean±SD)	65.7±10. 5	68.3±9.2	*0.036 ^b^*
Biochemical indicators (mean±SD)			
Glucose (mmol/l)	5.73±2.03	5.37±1.755	*0.165 ^b^*
Triglyceride (mmol/l)	1.71±1.05	1.53±0.82	*0.186 ^b^*
Cholesterol (mmol/l)	4.37±1.09	4.98±114	*< 0.001^b^*
HDL-C (mmol/l)	0.95±0.21	1.22±0.52	*< 0.001^b^*
LDL-C (mmol/l)	2.77±1.02	3.15±0.88	*0.006^b^*
ApoA (g/L)	1.22±0.24	1.25±0.30	*0.498 ^b^*
ApoB (g/L)	0.89±0.26	0.90±0.22	*0.785 ^b^*
CK-MB (IU/L)	48.63±78.45	12.50±6.62	*< 0.001 ^b^*

^a^Yates’ continuity corrected chi-square test. ^b^Unpaired t test with Welch's correction. HDL-C, high density lipoprotein cholesterol; LDL-C, low density lipoprotein cholesterol; CK-MB, creatine kinase-muscle/brain; ApoA, apolipoprotein A; ApoB, apolipoprotein B.

**Table 2:** Comparison of plasma levels of MEF2A in different groups or sub-groups.

**Groups/sub-groups**	**Number of samples**	**Mean MEF2A levels (ng/ml)**	**SD**	**Group Median**	**Statistics**	***p*-value**
CAD	118	0.91	0.42	0.78	-2.939^a^	0.003
Control	76	1.14	0.58	1.02		
**Acording to gender**						
Male CAD	80	0.95	0.45	0.85	-3.493^a^	< 0.001
Male control	27	1.42	0.72	1.14		
Female CAD	38	0.83	0.34	0.72	-1.797^a^	0.072
Female control	49	0.98	0.42	0.95		
**Acording to disease severity**						
Myocardiac infarction (MI)	70	0.89	0.44	0.75	2.109^b^	0.348
Unstable angina (UA)	42	0.93	0.40	0.87		
stable angina (SA)	6	1.04	0.37	1.15		
**Acording to age**						
<70y control	37	1.31	0.69	1.14	8.890^b^	0.031
≥70y control	39	0.98	0.40	0.98		
<70y CAD	71	0.85	0.37	0.75		
≥70y CAD	47	1.00	0.49	0.88		

Note: ^a^Wilcoxon rank sum test, z statistics; ^b^Kruskal Wallis test, Chi-Square statistics; SD, standard deviation.

Pearson correlation analysis showed that the plasma level of MEF2A was negatively correlated with age in the normal control group (*R* = -0.241, *P* = 0.03) (Supplementary Figure 1), and was negatively correlated with CAD after stratifying by age and gender (*R* = -0.286, *P* < 0.001) (Supplementary Figure 1). To eliminate the influence of the difference in the baseline data of the two groups on the analysis results, we used binary logistic regression analysis. CAD was used as a dependent variable, whereas age, gender, smoking, diabetes, MEF2A, total cholesterol, HDL-C, LDL-C, and CK-MB were included as covariates. The forward stepwise (likelihood ratio) method was used to carry out the regression process. Results showed that after excluding the above confounding factors, the plasma MEF2A levels were still significantly correlated with CAD (*P* = 0.016; *OR* = 0.359; 95% *CI*: 0.157 - 0.823) (Table 3). The above results suggested that the individuals with low plasma MEF2A level have a significantly increased risk of coronary heart disease.

**Table 3:** The variables were eventually included in the regression equation after logistic regression analysis.

**Indicators**	***B***	***SE***	***Odds ratio (OR)***	***P***	**95.0% C.I. for *OR***	
					**Lower**	**Upper**
MEF2A	–1.024	0.423	0.359	0.016	0.157	0.823
HDL-C	–2.215	0.676	0.109	0.001	0.029	0.411
LDL-C	–0.535	0.198	0.586	0.007	0.397	0.863
CK-MB	0.064	0.023	1.066	0.006	1.1019	1.116
Smoking	1.164	0.457	3.204	0.011	1.309	7.841
Constant	3.985	1.040	53.792	< 0.001		

Note: 95.0% C.I., 95% Confidence interval. *B*, Regression coefficients; SE, Standard error.

## DISCUSSION

We reported for the first time that MEF2A directly up-regulated the expression of PI3K (PIK3CA and PIK3CG) and activated its downstream signaling pathway. The up-regulation of PI3K/p-Akt/SIRT1 mediated the role of MEF2A in delaying HUVEC senescence. PI3K plays a key role in cell proliferation, survival, and differentiation and activates multiple signaling pathways [26]. MEF2A is also considered to play an important role in cell proliferation, survival, differentiation, and development [25]. However, the regulatory mechanism of its participation in cell life is unclear. This study found that MEF2A can directly regulate PI3K gene expression, suggesting that MEF2A may be involved in cell proliferation, survival, and differentiation and other life processes possibly through the PI3K/p-Akt signaling pathway. Chen et al. [27] showed that MEF2A inhibition can attenuate high-glucose-induced extracellular matrix accumulation by blocking the activation of the Akt and TGF-β1/Smad signaling pathways in cardiac fibroblasts, which also supports the activation role of MEF2A in the Akt signaling pathway. SIRT1 reportedly has protective functions in blood vessels, and its overexpression in the endothelium prevents cell senescence, enhances vasodilation, and reduces vascular damage caused by aging [28–30]. The regulation of the PI3K-Akt signaling pathway and SIRT1 has been extensively studied. Tomoaki et al. [31] found that the PI3K-Akt-GSK3β signaling pathway is required for endoplasmic reticulum stress-induced SIRT1 expression. Ming et al. [32] found that visfatin up-regulates SIRT1 expression via the PI3K/Akt/ERK pathway, thereby inhibiting ox-LDL-induced endothelial progenitor cell senescence. Resveratrol treatment combined with exercise training can improve the cardiac function of aged rats by increasing the activity of PI3K-Akt and SIRT1 [33]. These studies indirectly supported the findings that MEF2A increased SIRT1 gene expression by up-regulating the PI3K gene. However, Li et al. [34] found that SIRT1 promoted the migration and proliferation of mouse-spleen-derived endothelial progenitor cells by activating the PI3K/Akt/eNOS pathway. Chen et al. [7] further observed that protein kinase B2 (AKT2) deficiency in rat delayed cardiac development and decreased the expression of MEF2A, suggesting that a mutual regulatory loop may be present between the PI3K/Akt signaling pathway and SIRT1 or MEF2A.

After treating HUVECs with H_2_O_2_, MEF2A, PIK3CA, PIK3CG, p-AKT, and SIRT1 were significantly down-regulated but MEF2A overexpression in HUVECs prevented H_2_O_2_-induced decrease in PIK3CA, PIK3CG, p-AKT, and SIRT1. Thus, the oxidative-stress-induced decrease in SIRT1 was due to the oxidative stress down-regulating the expression of MEF2A. The oxidative-stress-induced downregulation of SIRT1 is one of the main molecular mechanisms underlying oxidative-stress-induced cell senescence. Hydrogen sulfide and curcumin prevent the H_2_O_2_-induced senescence of HUVECs by relying on the up-regulation of SIRT1 expression [35, 36]. The above studies suggest that the inhibition of the MEF2A/PI3K/SIRT1 pathway is one of the main causes of oxidative-stress-induced VEC senescence. Promoting the expression of MEF2A may be a new way to prevent VEC senescence. Zhou et al. [19] found that the inhibition of MEF2A by siRNA accelerated atherosclerosis in apoE-/- mice. The findings that MEF2A inhibition induced VEC senescence may explain to some extent the molecular mechanism of MEF2A knockdown to accelerate atherosclerosis.

We also found that the plasma MEF2A level was negatively correlated with CAD and age in the present study, which further indicated the importance of normal expression of MEF2A in the prevention of cardiovascular diseases and other senile diseases. In the logistic-regression equation, the regression coefficient of the plasma MEF2A levels was -1.024 and the odds ratio was 0.356, suggesting that high plasma MEF2A levels can greatly reduce the risk of CAD. In the normal control population, the plasma level of MEF2A was negatively correlated with age, whereas in the CAD patients, the plasma level of MEF2A was not correlated with age. Thus, the increased risk of CAD caused by the decrease in plasma MEF2A level did not depend on the increase in age. Moreover, the plasma MEF2A level may be a new potential biomarker for the risk prediction of CAD.

MEF2A is widely recognized for promoting skeletal muscle and cardiomyocyte proliferation, but few reports on the functional roles of MEF2A in cell senescence exist. Zhao et al. [21] reported that MEF2A promotes smooth muscle cell senescence by up-regulating microRNA-143/145, which is a potential molecular mechanism of the hydrogen peroxide-induced senescence of smooth muscle cells. Zhao et al. found that MEF2A promotes cell senescence in smooth muscle cells, which is contrary to the effect of MEF2A on the inhibition of HUVEC senescence in the present study. In fact, the different roles of MEF2A in various cells have been extensively reported. Some studies suggest that MEF2A promotes cell proliferation [37, 38], but reports that MEF2A promotes apoptosis also exist [39, 40]. The discrepancy may be explained by the fact that the different domains of MEF2A determine their nuclear localization and cell-type-specific transcriptional activity [41]. The differential splicing of MEF2A in cells of different tissue origins is also one of the reasons for the discrepancy. The differential splicing of MEF2A in the same tissue may even lead to a different disease status. Bachinski et al. [42] found significant differences in the splicing of MEF2A and MEF2C in muscle tissue between patients with myotonic dystrophy, neuromuscular disorders and normal people. Therefore, the distinct roles of MEF2A in SMC and VEC may attribute to the differential splicing of MEF2A.

Wang et al. [9] first reported that the functional deletion mutation of MEF2A is closely related to early-onset coronary heart disease. Since then, researchers have conducted a large number of studies on the relationship between the genetic polymorphism of MEF2A and coronary heart disease, but the findings are controversial. The relationship between MEF2A and vascular diseases such as CAD has not yet been determined. In the present study, we found that siRNA interference or the H_2_O_2_-induced downregulation of MEF2A can accelerate VEC senescence. One of the molecular mechanisms is to down-regulate the expression of SIRT1 by directly decreasing PI3K (PIK3CA and PIK3CG) expression. The biological age of endothelial cells in patients with cardiovascular disease is greater than their actual age. Endothelial aging exists in the early stages of vascular aging and contributes to the development of endothelial dysfunction and arterial stiffness. Functional defects in SIRT1 lead to premature cardiovascular and cerebrovascular diseases, metabolic disorders, diabetes, and neurodegenerative diseases [29, 30]. Therefore, our findings revealed the potential role and molecular mechanism of MEF2A in cardiovascular disease and provided a new perspective for further research on the pathological mechanism and prevention of senile diseases such as cardiovascular diseases.

In conclusion, the involvement of MEF2A in cardiocerebrovascular diseases may be related to the down-regulation of MEF2A expression in VECs. The molecular mechanism of delaying effect of MEF2A on VEC senescence involved the up-regulation of SIRT1 through the PI3K/p-Akt signal pathway. The decrease in plasma MEF2A level greatly increased the risk of CAD, and the plasma level of MEF2A may be a potential biomarker for predicting the risk of senile diseases such as CAD.

## MATERIALS AND METHODS

### Reagents

MEF2A-specific siRNA (SI-MEF2A: 5′-GGGCAGUUAUCUCAGGGUUT T-3′), negative control siRNA (5′-UUCUCCGAACGUGUCACGUTT-3′), and all PCR primers were synthesized by Sangon Biotech Co., Ltd. All primer sequences used are listed in Table 4. The mRNA quantitative reverse transcriptase PCR detection kit and RealUniversal color premix (SYBR Green) were purchased from Tiangen Biotech (Beijing) Co., Ltd. The dual luciferase reporter assay system was from Promega (Madison, WI, USA). The Pierce Agarose chromosome immunoprecipitation (ChIP) kit was from ThermoFisher Scientific. Antibodies against MEF2A and PI3KCG were purchased from FineTest (Wuhan, China). The antibodies against PI3KCA, AKT, p-AKT, SIRT1, p-P53, and beta-actin were purchased from Cell Signaling Technology (Beverly, MA, USA). The PI3K inhibitors AS-252424 and HS-173 were purchased from Selleck.

**Table 4:** Primer pairs for real-time quantitative PCR.

**Genes**	**Forward primer(5'to3')**	**Reverse primer (5'to3')**
MEF2A	AGCAGCCCTCAGCTCTCTTG	GGTGAAATCGGTTCGGACTTG
PIK3CG	TCCTCTTTGTGATGGGAACTT	TGTGTGATGACGAAGGGCT
PIK3CA	AGATAACTGAGAAAATGAAAGCTCACTCT	TGTTCATGGATTGTGCAATTCC
SIRT1	CTTCCCTCAAAGTAAGACCAG	ATTATGACATCACAGTCTCCAA
ACTB	GCACCACACCTTCTACAATG	TGGGGTGTTGATGGTCTC

### Cell culture

HUVECs were isolated from human umbilical vein and cultured in M199 medium (Gibco, USA) supplemented with growth factors, 5% fetal bovine serum (FBS), and double antibiotic (streptomycin and penicillin) at 37 °C in a 5% CO_2_ humid incubator. 293T cells were cultured in DMEM (Gibco, USA) supplemented with 10% FBS and 1% double antibiotic in a humid incubator of 5% CO_2_ at 37 °C.

### Vector construction

The complete MEF2A coding region fragment was amplified using high-fidelity PCR polymerase and cDNA template derived from HUVECs, and the amplified fragment was purified by gel extraction and then ligated into the mammalian expression vector pc3-gab at the *EcoR* I and *Bgl* II sites. The PIK3CA promoter (-1827 bp to 8 bp) region and the PIK3CG promoter (-1602 bp to 134 bp) region were amplified by PCR. The amplified DNA fragment was purified by gel extraction and then ligated into the restriction enzyme sites *Kpn* I and *Bgl* II located upstream of the Luciferase gene in the pGL3.0 basic vector (Promega, USA). Afterwards, transformation and plasmid extraction were performed, and the sequencing-verified plasmids were used in subsequent experiments.

### Transfection

Electroporation transfection: When HUVECs reached 85%–90% confluence, the cells were digested with trypsin containing 0.25% EDTA, and the supernatant was removed by centrifugation, resuspended in OPTI-MEM containing 5% FBS, and then diluted to a cell suspension of 1 × 10^6^ cells/ml. About 300 μl of cell suspension was dispensed into electric cups (electrode spacing = 4 mm). The prepared plasmids or siRNAs were added to the electric cups, mixed thoroughly, and stored at 4 °C for 5 min. Electric shock was performed on an electroporator (Gene Pulser Xcell 165-2660) according to the following conditions: square-wave model was used to pulse at 180 V twice for 40 ms and at a gap of 1 min between two pulses). Immediately after completion of the electric shock, the cell suspension was transferred to the wells containing complete growth medium, mixed thoroughly, and stored at 37°C and 5% CO_2_ in a humidified incubator. After 6 h, the medium was replaced with a fresh one, and culturing was performed for enough time according to the subsequent experiment needs.

The plasmids and siRNAs were transfected into cells using Lipofectamine 3000 or Lipofectamine RNAiMax according to the manufacturer’s instructions of the kit.

### RNA extraction and real-time fluorescence quantitative PCR analysis

Total RNA was extracted from cells using TRIzol reagent (Invitrogen, Carlsbad, CA, USA) and the first cDNA was synthesized using Fastking gDNA dispelling RT supermix according to the manufacturer’s protocol. The mRNA level of the genes was detected by real-time fluorescence quantitative PCR analysis with RealUniversal color premix kit (SYBR Green) according to the manufacturer’s instructions.

### Western blotting

Cells were washed with iced PBS and then lysed in RIPA buffer containing proteinase inhibitor. Protein content was measured with a BCA protein assay kit (Thermo, USA). Afterwards, an equal amount of protein samples (10 μg) was loaded onto 10% sodium dodecyl sulfate (SDS)–polyacrylamide gel for electrophoresis. The isolated proteins in gel were transferred to polyvinylidene fluoride membranes (Millipore, MA, USA). After blocking in 5% skim milk in TBST (10 mM Tris-HCl, 150 mM NaCl, and 0.1% Tween-20 (pH 8.0)) for 1 h at room temperature, the membranes were incubated with primary antibody overnight at 4°C. After thoroughly rinsing with TBST, the membranes were incubated with anti-IgG solution for 1 h at 37°C. Finally, a super chemiluminescence kit (KeyGen Biotech) was added to magnify the HRP signals, which were imaged on a multifunctional chemiluminescence imaging system (UVItec/ Alliance mini HD9).

### Luciferase activity analysis

The promoter vector and the Renilla luciferase vector (pRL-sv40) were co-transfected into 293T cells or the promoter vector, MEF2A over-expression vector and pRL-sv40 were co-transfected into 293T cells. At 48 h after transfection, the cells were treated according to the manufacturer’s instructions of the Dual Luciferase Reporter Assay system, and luciferase activity was measured for each well on a test tube luminometer (Lumat3 LB9508, Berthold).

### ChIP assay

ChIP assays were performed using the ChIP Assay Kit according to the manufacturer’s instructions. In a typical procedure, when the cells reached 90% confluence, they were treated with formaldehyde at a final concentration of 1% (v/v) for 10 min at 37 °C. Subsequently, the cells were harvested by centrifugation at room temperature for 5 min at 3000 g and lysed in 100 μl of SDS lysis buffer (1% SDS, 10 mM EDTA, and 50 mM Tri-HCl (pH 8.1)). Chromatin sonication was performed to shear the DNA to an average length of 200–1000 bp, followed by immunoprecipitation with the antibody against MEF2A and IgG. According to the CHIP kit instructions, 5 μl was collected from the 50 μl total sample as INPUT, and the remaining 45 μl was used for the immunoprecipitation experiment. The precipitated DNA was extracted and subjected to PCR amplification using the primer pairs spanning the potential MEF2A binding site in the PIK3CA and PI3KCG promoter regions.

### Cell viability assay

Cell viability was tested with MTS kit (CellTiter 96^®^ Aqueous one solution cell proliferation assay, Promega, USA) according to the manufacturer’s instruction. In a typical procedure, cells were seeded onto 96-well plates at a density of 6×10^3^ cells/well. After adherence overnight, various treatments were performed according to the experimental needs. The cells to be detected in the 96-well plate were washed with PBS (pH 7.2) and 10 μl of MTS solution was added, followed by incubation for 3 h at 37 °C. Then, the absorbance at 490 nm was measured on a 96-well plate reader. Relative cell viability was expressed as the fold changes relative to the control group.

### Senescence associated β-galactosidase (SA-β-Gal) assay

SA-β-Gal activity was measured with a senescence β-Galactosidase Staining Kit (CST, USA) according to the kit instructions. In a typical procedure, cells were washed with PBS and fixed with 0.5% glutaraldehyde for 15 min at room temperature. The cells were washed again with PBS twice, and SA-β-Gal stain solution was added before incubating at 37 °C in darkness. Cells that were positive for SA-β-Gal staining were examined under a light microscope (Nikon Ti-U, Japan).

### Participants

Both the case group and the control group in this study were selected from inpatients in the Department of Cardiology, the Second Affiliated Hospital of Guangzhou Medical University from 2012 to 2015. The selection criteria of CAD were as follows: coronary angiography showed at least one main coronary artery stenosis ≥50%, or patients with a history of coronary heart disease or acute myocardial infarction, and the age was <85 years old. Exclusion criteria: Individuals with cardiomyopathy, congenital heart disease, liver failure, renal failure, hepatitis, hemorrhagic diseases, tumors, and other malignant diseases were excluded. A total of 118 CAD patients were included in the final case group.

The selection criteria of the controls were as follows: male ≥ 55 years old, female ≥ 60 years old, no stenosis of the main coronary artery or stenosis of at most one main branch of the coronary artery and the degree of stenosis should be less than 50%, or no history of chest pain in patients without angiography. Exclusion criteria: Individuals with cardiomyopathy, valvular disease, congenital heart disease, stroke, venous thrombosis, liver failure, renal failure, hepatitis, hemorrhagic diseases, tumors, and other malignant diseases were excluded. Patients with rheumatoid, lupus erythematosus, and other serious autoimmune diseases were also excluded. The final control group included 76 participants.

All subjects underwent biochemical examinations, including those for blood glucose, total triglyceride, total cholesterol, high-density lipoprotein cholesterol (HDL-C), and low-density lipoprotein cholesterol (LDL-C), apolipoprotein A (ApoA), apolipoprotein B (ApoB), and creatine kinase-muscle/brain (CK-MB). These biochemical indicators were detected by the Department of Clinical Laboratory, the Second Affiliated Hospital of Guangzhou Medical University. All participants were Han Chinese from Southern China and all gave written informed consent. The Institute Research Medical Ethics Committee of Guangzhou Medical University approved this study.

### Enzyme-linked immunosorbent assay (ELISA)

The plasma levels of MEF2A were tested with a human MEF2A (myocyte enhancer factor 2A) ELISA kit (FineTest, Wuhan, China). Plasma was separated from whole blood samples using EDTA-K_2_ as an anticoagulant. The whole blood samples were centrifuged for 15 min at 1000×g at 2–8°C within 30 min of collection. The supernatant was collected and the assay was conducted according to the manufacturer’s instruction for the ELISA kit.

### Statistical analysis

The expression levels of each gene in the treatment and control groups were normalized to beta-actin and then divided by its value in the control group to obtain a fold changes relative to the control group. Three or more independent experiments were performed for each experimental treatment, and the relative expression levels of the genes were expressed as the mean ± standard error. Firefly luciferase activity was normalized with Renilla luciferase activity and then divided by its value in the control group, expressed as the mean ± standard error of more than three independent experiments. The significance of the difference between the two groups was examined by Student’s t test or two-way ANOVA with bonferroni post tests, and one-way ANOVA was used to test the significance in multiple sets of comparison. Detection of Variance homogeneity of data in each Group or subgroup was performed by using exploratory analysis in descriptive analysis in SPSS16 software. For the significance test of difference between two groups or two subgroups with uneven variance, Wilcoxon rank sum test (Equivalent to Mann-Whitney U test) in nonparametric test in SPSS16 software was used. For the significance test of difference between multiple groups or subgroups with uneven variance, Kruskal-Wallis H test in nonparametric test in SPSS16 software was used. The difference in gender distribution between patients and controls was assessed with Yates’ continuity corrected chi-square test. Unpaired t test with Welch's correction in GraphPad Prism V5.01 software was used for significant difference detection between two groups of data with uniform Variance. Binary logistic regression and Pearson correlation analysis were performed by using SPSS version 16. The difference was considered significant when p < 0.05.

## Supplementary Material

Supplementary Figure
