# Supplementary material for: Myocyte enhancer factor 2A delays vascular endothelial cell senescence by activating the PI3K/p-Akt/SIRT1 pathway
Source: Aging (Albany NY). 2019 Jun 10;11(11):3768–84. doi: 10.18632/aging.102015 (PMC6594820; doi:10.18632/aging.102015)
Supplement: Supplementary Figure [file aging-11-102015-s001.pdf]

SUPPLEMENTARY FIGURE

Descriptive Statistics

	Mean	Std. Deviation	N
MEF2A	.999983	.5032107	194
CAD	.608247	.4894049	194
age	6.6979E1	10.2380772	194
Gender	1.4484E0	.4986226	194

Correlations

Control Variables			MEF2A	CAD	age	Gender
-none-[a]	MEF2A	Correlation	1.000	-.221	-.014	-.154
		Significance (2-tailed)	.	.002	.841	.032
		df	0	192	192	192
	CAD	Correlation	-.221	1.000	-.166	-.317
		Significance (2-tailed)	.002	.	.021	.000
		df	192	0	192	192
	age	Correlation	-.014	-.166	1.000	.322
		Significance (2-tailed)	.841	.021	.	.000
		df	192	192	0	192
	Gender	Correlation	-.154	-.317	.322	1.000
		Significance (2-tailed)	.032	.000	.000	.
		df	192	192	192	0
age & Gender	MEF2A	Correlation	1.000	-.286		
		Significance (2-tailed)	.	.000		
		df	0	190		
	CAD	Correlation	-.286	1.000		
		Significance (2-tailed)	.000	.		
		df	190	0		

a. Cells contain zero-order (Pearson) correlations.

Supplementary Figure 1. Pearson correlation analysis for CAD and plasma MEF2A level by controlling age and gender.
